# Degradation and Photocatalytic Properties of Nanocomposites

**DOI:** 10.3390/nano14131065

**Published:** 2024-06-21

**Authors:** Peter Kasak

**Affiliations:** Center for Advanced Materials, Qatar University, Doha 2713, Qatar; peter.kasak@qu.edu.qa

## 1. Introduction

The world is suffering from energy consumption and environmental pollution challenges for the next generation era. The developments in the construction of sustainable resources to overcome the energy crises have been made in several ways to compete with the depletion of fossil fuels. Among those, photocatalytic hydrogen production has proven to be an alternative for sustainable and green energy production pathways in nanoscience and technologies. Thus, hydrogen production technologies are renowned for renewable feedstock and greenhouse gas-free technology [1,2,3]. Since the technology has been introduced, several strategies have been adopted to develop efficient catalyst materials to be employed for splitting water molecules for hydrogen production under solar light [4].

The fundamental engineering for tuning the nanocomposite materials in photocatalytic applications requires special features of the catalyst materials to be addressed. Among those, the selection of materials with cheap, earth-abundant, and environmentally friendly characteristics may apply to large-scale industrial projects with state-of-the-art enhanced performance [5,6]. For designing such nanocomposite materials, carbon and carbon-based 2D materials combined with metal oxides are considered significant achievements in energy and environmental applications due to the enhanced surface area and possible intra-junctions of the catalyst materials. These specific features of the nanocomposite materials set footprints for developing more efficient photocatalyst materials for broader sustainable energy applications, i.e., photo-(electro)catalytic green fuel production [7,8,9,10].

Towards environmental concerns, various strategies have been used to deal with environmental pollution issues by converting pollutants by degrading them completely into less hazardous species [11,12,13,14]. Similar to the above-mentioned photocatalytic applications, the design of materials for photodegradation also works on the concept of band structures combined in developing heterojunctions, which highly improves the charge separation efficiency and redox behavior of the photodegradation materials due to the photo-induced phenomenon [15,16].

## 2. An Overview of Published Articles

Cheng et al. (Contribution 1) introduced a mixed metal oxide W-TiO_2_ nanopowder photocatalyst by using the sol–gel method, varying the compositions of each component in the catalyst material. The synthesized catalyst was tested for the photocatalytic removal capacity of a representative pollutant, methylene blue (MB), in aqueous solutions and under UV-A and sunlight illuminations. They ascribed the enhanced performance of the material to the combined action of adsorption and heterogeneous photocatalysis. 

Bolaghi et al. (Contribution 2) synthesized and reported the graphitic carbon nitride (g-C_3_N_4_), a metal-free photocatalyst for multi-photocatalytic applications such as visible-driven hydrogen production, CO_2_ reduction, and organic pollutant degradation. They mainly focused on and were convinced of the approaches of photochemical stability, cost-effectiveness, and scalable synthesis of the photocatalysts. They also proposed that the photocatalytic performance of catalysts following ultrasonication prevents the agglomeration of g-C_3_N_4_ nanosheets and also tunes pore size distribution, which plays a crucial role in the high performance of the catalyst material.

Yousaf et al. (Contribution 3) introduced and reported a heteronanostuctured photocatalyst comprising g-C_3_N_4_ coupled with ZnCdS for photocatalytic hydrogen production applications. The enhanced and durable performance of the catalyst for hydrogen production in their work was ascribed to the heterojunction formation established among two components and the resulting synergistic effect, which provided more channels for charge carrier migration and reduced the recombination of photogenerated electrons and holes.

Mbuyazi and Ajibade (Contribution 4) published and discussed the role of different capping agents influencing the structural, optical, and photocatalytic degradation efficiency of the magnetite (Fe_3_O_4_) nanoparticles. In their work, octylamine (OTA), 1-dodecanethiol (DDT), and tri-n-octylphosphine (TOP) different capping agents were used for the synthesis of magnetite nanoparticles. Among these, tri-n-octylphosphine-capped iron oxide nanoparticles proved to be the most efficient iron oxide nanophotocatalysts for the degradation of the dyes.

Song et al. (Contribution 5) worked on semiconductor photocatalyst materials in the field of environmental remediation. They reported that BiOCl-based ternary photocatalysts can be used for the applications of photocatalytic degradation of highly toxic norfloxacin. They convinced that the higher crystallinity of BiOCl closely aligned molecules with each other, which was the major cause of their improved separation efficiency of photogenerated charges and showed high degradation efficiency for norfloxacin antibiotics.

Zeng et al. (Contribution 6) report the synthesis of Ag/P25 nanocomposites through a one-step gamma-ray radiation method and tested them for the applications of photocatalytic degradation of organic contaminations. In their findings, it is proposed that the particle size of Ag could be effectively controlled by changing the dose rate, and the Ag/P25 nanocomposites doped with smaller Ag nanoparticles performed higher photocatalytic activities.

Zavahir et al. (Contribution 7) propose a titanium nanotube array (based on a non-ferrous Fenton system) for the successful degradation of a model contaminant, the azo dye methyl orange, under simulated solar illumination. Their contributions proposed that the facile withdrawal and regeneration observed in the film-based titanium nanotube array photocatalyst highlight its potential to treat real industrial wastewater streams.

Islam et al. (Contribution 8) worked on a track for the synthesis of spherical silver nanoparticles (AgNPs), carbon nanospheres (CNSs), and a bispherical AgNP–CNS nanocomposite by the facile thermal procedure. The as-synthesized material was employed for photo-degradation of organic dyes. In their detailed reported observations, it was found that in the AgNP–CNS nanocomposite, the light absorption and UV utilization capacity increased at more active sites. Moreover, the effective electron-hole separation at the heterojunction between the AgNPs and CNSs was possible under favorable band-edge conditions, resulting in the creation of reactive oxygen species.

Xu et al. (Contribution 9) developed the construction of direct Z-scheme heterojunctions-based catalyst material for the applications of photoreduction of CO_2_ to 100% alcohol products. They claimed that the combination of Bi_2_WO_6_ and SnS_2_ narrows the bandgap, thereby broadening the absorption edge and increasing the absorption intensity of visible light. These specified characteristics enhanced the performance of the reported catalyst.

## 3. Conclusions

In conclusion, the articles that contributed to this Special Issue focused on state-of-the-art synthesis and modifications in photocatalytic materials. The combined photocatalytic applications considered were mainly based on energy and environmental challenges. Regarding energy-related topics, photocatalytic hydrogen production and CO_2_ reduction were enlisted and tested with developed materials. The advancements in the development of catalyst materials for these energy applications may provide extensive advantages for nanoscience and technology inputs in sustainable energy resources. 

In addition, the focus on developing material for photodegradation of hazardous pollutant molecules was also devoted to this issue. The efficient material with scalable synthesis and enhanced photodegradation of organic pollutants may significantly contribute to the environmental applications with straightforward material synthesis approaches. The possible solutions for both the energy and environmental challenges considered in this Special Issue positively impact modern and next-generation society. 

## 4. List of Contributions

1. Cheng, K.; Heng, S.; Tieng, S.; David, F.; Dine, S.; Haddad, O.; Colbeau-Justin, C.; Traore, M.; Kanaev, A. Mixed Metal Oxide W-TiO_2_ Nanopowder for Environmental Process: Synergy of Adsorption and Photocatalysis. *Nanomaterials* **2024**, *14*, 765. https://doi.org/10.3390/nano14090765.
2. Kalantari Bolaghi, Z.; Rodriguez-Seco, C.; Yurtsever, A.; Ma, D. Exploring the Remarkably High Photocatalytic Efficiency of Ultra-Thin Porous Graphitic Carbon Nitride Nanosheets. *Nanomaterials* **2024**, *14*, 103. https://doi.org/10.3390/nano14010103.
3. Yousaf, A.B.; Imran, M.; Farooq, M.; Kausar, S.; Yasmeen, S.; Kasak, P. Graphitic Carbon Nitride Nanosheets Decorated with Zinc-Cadmium Sulfide for Type-II Heterojunctions for Photocatalytic Hydrogen Production. *Nanomaterials* **2023**, *13*, 2609. https://doi.org/10.3390/nano13182609.
4. Mbuyazi, T.B.; Ajibade, P.A. Influence of Different Capping Agents on the Structural, Optical, and Photocatalytic Degradation Efficiency of Magnetite (Fe_3_O_4_) Nanoparticles. *Nanomaterials* **2023**, *13*, 2067. https://doi.org/10.3390/nano13142067.
5. Song, D.; Li, M.; Liao, L.; Guo, L.; Liu, H.; Wang, B.; Li, Z. High-Crystallinity BiOCl Nanosheets as Efficient Photocatalysts for Norfloxacin Antibiotic Degradation. *Nanomaterials* **2023**, *13*, 1841. https://doi.org/10.3390/nano13121841.
6. Zeng, Z.; Li, S.; Que, X.; Peng, J.; Li, J.; Zhai, M. Gamma Radiation Synthesis of Ag/P25 Nanocomposites for Efficient Photocatalytic Degradation of Organic Contaminant. *Nanomaterials* **2023**, *13*, 1666. https://doi.org/10.3390/nano13101666.
7. Zavahir, S.; Elmakki, T.; Ismail, N.; Gulied, M.; Park, H.; Han, D.S. Degradation of Organic Methyl Orange (MO) Dye Using a Photocatalyzed Non-Ferrous Fenton Reaction. *Nanomaterials* **2023**, *13*, 639. https://doi.org/10.3390/nano13040639.
8. Islam, M.A.; Akter, J.; Lee, I.; Shrestha, S.; Pandey, A.; Gyawali, N.; Hossain, M.M.; Hanif, M.A.; Jang, S.G.; Hahn, J.R. Facile Preparation of a Bispherical Silver–Carbon Photocatalyst and Its Enhanced Degradation Efficiency of Methylene Blue, Rhodamine B, and Methyl Orange under UV Light. *Nanomaterials* **2022**, *12*, 3959. https://doi.org/10.3390/nano12223959.
9. Xu, Y.; Yu, J.; Long, J.; Tu, L.; Dai, W.; Yang, L. Z-Scheme Heterojunction of SnS_2_/Bi_2_WO_6_ for Photoreduction of CO_2_ to 100% Alcohol Products by Promoting the Separation of Photogenerated Charges. *Nanomaterials* **2022**, *12*, 2030. https://doi.org/10.3390/nano12122030.
